# Multiclass relevance units machine: benchmark evaluation and application to small ncRNA discovery

**DOI:** 10.1186/1471-2164-14-S2-S6

**Published:** 2013-02-15

**Authors:** Mark Menor, Kyungim Baek, Guylaine Poisson

**Affiliations:** 1 Department of Information and Computer Sciences, University of Hawai`i at Mānoa, Honolulu, HI 96822, USA

## Abstract

**Background:**

Classification is the problem of assigning each input object to one of a finite number of classes. This problem has been extensively studied in machine learning and statistics, and there are numerous applications to bioinformatics as well as many other fields. Building a multiclass classifier has been a challenge, where the direct approach of altering the binary classification algorithm to accommodate more than two classes can be computationally too expensive. Hence the indirect approach of using binary decomposition has been commonly used, in which retrieving the class posterior probabilities from the set of binary posterior probabilities given by the individual binary classifiers has been a major issue.

**Methods:**

In this work, we present an extension of a recently introduced probabilistic kernel-based learning algorithm called the Classification Relevance Units Machine (CRUM) to the multiclass setting to increase its applicability. The extension is achieved under the error correcting output codes framework. The probabilistic outputs of the binary CRUMs are preserved using a proposed linear-time decoding algorithm, an alternative to the generalized Bradley-Terry (GBT) algorithm whose application to large-scale prediction settings is prohibited by its computational complexity. The resulting classifier is called the Multiclass Relevance Units Machine (McRUM).

**Results:**

The evaluation of McRUM on a variety of real small-scale benchmark datasets shows that our proposed Naïve decoding algorithm is computationally more efficient than the GBT algorithm while maintaining a similar level of predictive accuracy. Then a set of experiments on a larger scale dataset for small ncRNA classification have been conducted with Naïve McRUM and compared with the Gaussian and linear SVM. Although McRUM's predictive performance is slightly lower than the Gaussian SVM, the results show that the similar level of true positive rate can be achieved by sacrificing false positive rate slightly. Furthermore, McRUM is computationally more efficient than the SVM, which is an important factor for large-scale analysis.

**Conclusions:**

We have proposed McRUM, a multiclass extension of binary CRUM. McRUM with Naïve decoding algorithm is computationally efficient in run-time and its predictive performance is comparable to the well-known SVM, showing its potential in solving large-scale multiclass problems in bioinformatics and other fields of study.

## Background

The problem of classifying an object to one of a finite number of classes is a heavily studied problem in machine learning and statistics. There are numerous applications in bioinformatics, such as cancer classification using microarrays [1], prediction of protein localization sites [2,3], protein fold recognition [4], and identification of the kinase that acts upon a protein phosphorylation site [5].

Recently, a novel kernel-based learning algorithm called the Classification Relevance Units Machine (CRUM) for binary classification was introduced [6]. The CRUM addresses some of the concerns in the use of the Support Vector Machine (SVM) [7], including removing the specification of the error/complexity trade-off parameter by using empirical Bayes methods, generation of more parsimonious models, and providing probabilistic outputs through the estimation of the posterior probability density. Furthermore, the training algorithm is more efficient than that of the Relevance Vector Machine (RVM) [8] that similarly addressed the SVM concerns. The highly compact model the CRUM generates significantly reduces the run-time of the prediction system and hence provides further advantages over the SVM in conducting large-scale data analyses [9], such as Next Generation Sequencing (NGS) data analysis.

In this paper, we extend the CRUM algorithm into the more general multiclass setting, allowing for applications beyond binary classification. This is achieved by decomposing the multiclass problem into a set of binary classification problems using the error correcting output codes (ECOC) [10] framework. To preserve the probabilistic outputs of the binary CRUM into the multiclass setting, the algorithm based on the generalized Bradley-Terry (GBT) model [11] is considered. Since the optimization problem solved by the GBT algorithm can prohibit its use in large-scale classification settings, we also propose a simple linear-time algorithm as an alternative. The details of the Multiclass Relevance Units Machine (McRUM) construction based on the binary CRUM are described in the next section.

In this study, the McRUM is evaluated on two sets of experiments. First, the McRUM is applied to a variety of small-scale datasets from the UCI repository [12] in order to compare the performance of the McRUM under different settings by using different decompositions of the multiclass problem into a set of binary classification problems and the use of two different decoding algorithms that aggregate the binary predictions into multiclass predictions.

In the second set of experiments, the McRUM is applied to the problem of classifying small noncoding RNAs (ncRNAs) to validate the use of the method on a problem of a larger scale than that of the first set of experiments. This second set of experiments deal with a three-class classification problem, specifically, the identification of sequences from two classes of post-transcriptional gene regulatory ncRNAs - mature microRNA (miRNA) and piwi-interacting RNA (piRNA) - from other ncRNAs. This is of interest to small RNA sequencing projects (under 40 nt) where novel miRNAs and piRNAs can be found amidst a set of unannotated reads. For the miRNAs, it is especially interesting since the miRNA precursors may not be sequenced in those small ncRNA sequencing project, and thus losing the usual avenue of finding novel miRNAs via identification of their precursors [13]. Furthermore, the predictions with the McRUM are based solely on the RNA sequences and no additional genomic information is required, which is ideal for the study of organisms whose genomic information is lacking.

The experimental results on datasets taken from the UCI repository together with the preliminary results on small ncRNAs show that, under certain settings, the McRUM can achieve comparable or higher accuracy than previous analyses of these problems. Thus the results suggest CRUM's potential in solving multiclass problems in bioinformatics and other fields of study.

## Methods

### Classification relevance units machine

The sparse kernel-based binary classification model called the Classification Relevance Units Machine (CRUM) obtains probabilistic predictions [6,9]. Let Ψ to be a set of objects; e.g. Ψ ⊆ R*^d^*. The CRUM models the posterior distribution *p*(*C*_+_|**x**) that an object **x **∈ Ψ is a member of the positive class *C*_+ _using the following model

(1) $$P\left(C_{+}\;|x\right)=\sigma \left(\sum_{i=1}^{M}w_{i}k\left(x,u_{i}\right)+b\right)$$

where *σ *is the sigmoid function, *M *is a positive integer, *k*(·,·) is a kernel function, the weights *w_i _*∈ R, the bias *b *∈ R, and the Relevance Units (RUs) **u***_i _*∈ R*^d^*. The posterior of the negative class is then *P*(*C*_-_|**x**) = 1 - *P*(*C*_+_|**x**).

For a given *k*(·,·), *M*, an observed dataset *X *= {**x**_1_, **x**_2_,..., **x***_N_*} ⊆ Ψ and the associated class labels $\left\{c_{{\text{x}}_{\text{1}}},\;c_{{\text{x}}_{2}},...,c_{{\text{x}}_{N}}\right\}$, the binary CRUM learning algorithm first estimates the kernel parameter(s) and **u***_i_*'s through unsupervised learning, and then learns the values of the *w_i_*'s, and *b *through an iterative approach. The CRUM learning algorithm minimizes structural risk under log loss [7,14] and determines the error/complexity trade-off parameter using an empirical Bayes method. Further details can be found in [6,9].

### The multiclass classification problem and solutions

Multiclass classification is the generalization of binary classification to an arbitrary number of classes *K *> 1. We denote the set of *K *classes as *T *= {*C*_1_, *C*_2_,..., *C_K_*}, and want to learn a classifier function *g*: Ψ → *T*.

There are two major approaches to converting a binary classifier to a multiclass classifier: the direct approach and through the aggregation of multiple binary classifiers.

#### Direct approach

In the direct approach, the internals of the binary classifier are changed to reflect the *K *class situation. For CRUM, this is done by changing the underlying model from the binary sigmoid model to a multiclass softmax model,

(2) $$P\left(C_{j}|x\right)=\frac{exp\left(\sum_{m=1}^{M}w_{mj}k\left(x,u_{m}\right)+b_{j}\right)}{\sum_{i=1}^{K}exp\left(\sum_{m=1}^{M}w_{mi}k\left(x,u_{m}\right)+b_{i}\right)}$$

where the *M *RUs **u***_m_*, *M*·*K *weights *w_mi_*, and *K *biases *b_i _*are to be learned. The RUs can be learned using unsupervised learning on the unlabeled data, as done in the binary case [9]. The *K *times increase in parameters lead to a *K*^3 ^increase in the run-time complexity of the CRUM training algorithm compared to the binary case, due to the inversion of the (*M*·*K *+ 1) × (*M*·*K *+ 1) Hessian matrix. Similar to the RVM, this may make this method impractical for large problems [8]. Furthermore, related work in softmax regression suggests the need for more elaborate and costly methods for matrix inversion due to ill-conditioning [15].

Likewise, reformulating the SVM for multiclass classification leads to high cost training algorithms [16]. Therefore the second approach of aggregating multiple binary classifiers, which we will discuss next, has been the more popular and practical way to solve the multiclass classification problem.

#### Decomposition of a multiclass problem into binary classification problems

The idea of the aggregation approach is to decompose the multiclass problem into multiple binary problems that can then be solved with binary classifiers. The most popular framework for this approach is the method of error correcting output codes (ECOC) [10]. In this framework, the decomposition of a *K*-class problem into *L *binary problems is expressed with a coding matrix,

(3) $$M\in {\left\{0,1,\Delta \right\}}^{K\times L}$$

where each column of **M **specifies one binary classifier.

For example, the one-versus-rest (OVR) matrix for three classes is a 3 × 3 identity matrix:

(4) $$\left(\begin{array}{ccc} 1 & 0 & 0\\ 0 & 1 & 0\\ 0 & 0 & 1 \end{array}\right)$$

There are three columns and thus this decomposition will require the training of three binary classifiers. The first binary classifier is trained with the training data belonging to class *C*_1 _as the positive class set and the data belonging to classes *C*_2 _and *C*_3 _as the negative class set. The second binary classifier is trained with the training data belonging to class *C*_2 _as the positive class set and the data belonging to classes *C*_1 _and *C*_3 _as the negative set. The third binary classifier is trained similarly. The name of this decomposition is called one-versus-rest (OVR) because each binary classifier is trained with only one class serving as the positive class and all other classes serving as the negative class. In general, the OVR matrix for *K *classes is the *K *× *K *identity matrix.

The all-pairs (AP) matrix for three classes is also a 3 × 3 matrix:

(5) $$\left(\begin{array}{ccc} 1 & 1 & \Delta \\ 0 & \Delta & 1\\ \Delta & 0 & 0 \end{array}\right)$$

The Δ symbol denotes omission of the class in the training of the binary classifier. Therefore in this case, the first binary classifier is trained with the training data belonging to class *C*_1 _as the positive class set, data from *C*_2 _as the negative class set, and data from *C*_3 _is omitted. The next two binary classifiers are trained in a similar way. This decomposition is called one-versus-one or all-pairs (AP) as each binary classifier is trained with only a single class serving as the positive class and another single class as the negative class. Since there are *K*(*K *- 1)/2 distinct pairs of classes, the general AP matrix for *K *classes is a *K *× *K*(*K *- 1)/2 matrix.

In general any coding matrix **M **defined by Equation (3) can be used under the following constraints:

1. All rows and columns are unique

2. No row is solely composed of Δ

3. Each column has at least one 1 and 0

#### Aggregating the binary outputs

Given a coding matrix **M **and the outputs of the *L *binary classifiers, how do we compute the probability *P*(*C_k_*|**x**)? Let us first consider the simple case of hard decoding, leading to a hard decision. Assume that the binary classifiers *g_i_*, corresponding to the binary classifier specified by the *i*-th column of **M**, return hard decisions where an output of 1 denotes the positive class and 0 denotes the negative class. Then the collective output of the binary classifiers on **x **can be collected into a row vector **g**(**x**) = [*g*_1_(**x**), *g*_2_(**x**),..., *g_L_*(**x**)]. The predicted class that **x **belongs to is determined by finding the row of **M **with the smallest distance to **g**(**x**). Let **y**, **z **∈ {0, 1, Δ}^1 × *L*^*_. _*A commonly used measure of distance is a modified Hamming distance [17]:

(6) $$d\left(y,z\right)=\sum_{i=1}^{L}cost\left(y_{i},z_{i}\right)$$

where

(7) $$cost\left(a,b\right)=\left\{\begin{array}{cc} 0 & \text{if}\;a\;=\;b\;\text{and}\;a\neq \Delta \\ 1 & \text{if}\;a\;\neq \;b\;\text{and}\;a,b\neq \Delta \\ 1\;/2 & \text{if}\;a\;\text{or}\;b\;=\Delta \end{array}\right)$$

Let **M**(*k*) denote the *k*-th row of **M **and,

(8) $$k^{*}=\text{arg}{\text{min}}_{k\in \left\{1,2,...,K\right\}}d\left(M\left(k\right),g\left(x\right)\right)$$

Then the predicted class of **x **is $C_{k*}$. In the case of the AP coding matrices, this would correspond to choosing the class with the majority vote of the binary classifiers. Note that rows of **M **can be interpreted as the unique codewords representing the *K *classes and that the predicted **g**(**x**) is one of those codewords corrupted by noise. In this context, the above algorithm decodes **g**(**x**) into the closest codeword, thus performing error correction on the corrupted bits and giving the name of this approach to classification, ECOC.

Unfortunately, computing the posterior probabilities *p_k _*= *P*(*C_k_*|**x**) for all *K *classes is more difficult. For general coding matrices, the Generalized Bradley-Terry (GBT) model is used to estimate the posterior probabilities [11]. Let $I_{i}^{+}$ and $I_{i}^{-}$ denote the set of positive and negative classes, respectively, used by *g_i_*. Then the output *g_i_*(**x**) is the probability of the positive class of the *i*-th binary classification problem. Also let *N_i _*denote the number of training data with classes in $I_{i}=I_{i}^{+}\cup I_{i}^{-}$, and the following quantities:

(9) $$q_{i}=\sum_{k\in I_{i}}p_{k}$$

(10) $$q_{i}^{+}=\sum_{k\in I_{i}^{+}}p_{k}$$

(11) $$q_{i}^{-}=\sum_{k\in I_{i}^{-}}p_{k}$$

Given the probabilistic outputs of the binary classifiers ${\hat{r}}_{i}=g_{i}\left(x\right)$, the core idea of the GBT model is that

(12) $${\hat{r}}_{i}\approx P\left(I_{i}^{+}|x,I_{i}\right)=\frac{q_{i}^{+}}{q_{i}}$$

Through these relations the posterior probabilities **p **= [*p*_1_, *p*_2_,..., *p_K_*]*^T ^*can be retrieved. This is done by finding the **p **that minimizes the negative log-likelihood,

(13) $$-\sum_{i=1}^{L}N_{i}\left({\hat{r}}_{i}log\left(q_{i}^{+}/q_{i}\right)\;+\;\left(1-{\hat{r}}_{i}\right)log\left(q_{i}^{-}/q_{i}\right)\right)$$

under the constraints that each *p_k _*> 0 and that they sum to unity. This optimization can be interpreted as the minimization of the weighted Kullback-Leibler divergence between ${\hat{r}}_{i}$and $q_{i}^{+}/q_{i}$. Huang et al. [11] proposed an iterative algorithm to solve this optimization.

Note that the optimization of Equation (13) must be done for every object **x **that we want to make a prediction on. This could be too expensive in large-scale prediction applications. Furthermore, the computational complexity of the algorithm is not completely characterized. While Huang et al. [11] provide a proof of convergence under some assumptions, under a general decomposition the algorithm may not converge. In the cases that are known to converge, the speed of convergence is unknown. Therefore, a naive approach is proposed.

We make the naive assumption that the output of each binary classifier is independent. Under the interpretation of error-correcting codes, the formulation below is a soft-decoding of the observed **g**(**x**) to the codewords in **M **under the assumption that bit errors are independent. Then we can compute the class posteriors as simple products of the binary posteriors, as follows

$$P\left(C_{k}\;|x,M\right)=\overset{L}{\underset{i=1,M_{ki}\neq \Delta }{\prod }}P{\left(I_{i}^{+}|x,I_{i}\right)}^{M_{ki}}P{\left(I_{i}^{-}|x,I_{i}\right)}^{1-M_{ki}}$$

(14) $$\approx \prod_{i=1,M_{ki}\neq \Delta }^{L}g_{i}{\left(x\right)}^{M_{ki}}{\left(1-g_{i}\left(x\right)\right)}^{1-M_{ki}}$$

where the output of classifiers not trained on data from class *C_k _*are omitted. For example, from the decomposition given in Equation (5), *P*(*C*_2_|**x**, **M**) = (1 - *g*_1_(**x**))*g*_3_(**x**). Given the outputs of the binary classifiers, the algorithm is linear in *L*. In the implementation log of Equation (14) is used for computational stability as shown in Step 4 of Algorithm 2.

The above formulation is a generalization to any valid **M **of the Resemblance Model for AP decomposition proposed in [18]. Again, the key assumption is the independence of the *L *binary classifiers, which is highly dependent on the decomposition **M**. Thus in general, this method is possibly only a crude approximation.

The following pseudocodes summarize the training and prediction processes of McRUM.

**Algorithm 1: **Training McRUM

   Input: *K *× *L *decomposition matrix **M**, labeled training data

     1:  **for ***i *= 1 **to ***L*

     2:       *positive_data *← initialize as empty

     3:       *negative_data *← initialize as empty

     4:      **for ***j *= 1 **to ***K*

     5:         **if M***_ij _*= = 1

     6:             Add data from class *j *to *positive_data*

     7:        **else if M***_ij _*== 0

     8:          Add data from class *j *to *negative_data*

     9:        **end if**

     10:    **end for**

     11:    Set *g_i _*to binary CRUM trained on *positive_data *and *negative_data *as described in [9]

     12:    **end for**

     13:   **return g **= [*g*_1_, *g*_2_,..., *g_L_*]

**Algorithm 2: **Prediction

   Input: *K *× *L *decomposition matrix **M**, *L *binary CRUMs *g_i_*, input **x**

     1:  Set **p **= [*p*_1_, *p*_2_,..., *p_K_*] = [0, 0,..., 0]

     2:  **for ***i *= 1 **to ***K*

     3:       **for ***j *= 1 **to ***L*

     4.          $p_{i}=p_{i}+ln\left\{g_{j}{(x\text{)}}^{M_{ij}}{\left(1-g_{j}(x)\right)}^{\left(1-M_{ij}\right)}\right\}$

     5:        **end if**

     6:  **end for**

     7:  $p=\text{exp}\left(p\right)/)\sum_{}\text{exp}\left(p\right)$ (normalize to ensure the posterior probabilities sum to 1)

     8:  **return p**

#### Optimal coding matrix

The next question is whether there is any theory that can help guide us to designing the optimal coding matrix that gives the smallest error. There is, but it is not practically useful. These are some of the properties that would achieve a good ECOC-based classifier [17]:

1. The minimum distance (using Hamming distance, Equation (6)) between rows of **M **should be maximized

2. The number of **Δ **entries should be minimized

3. The average error of the *L *binary classifiers should be minimized

All the criteria are at odds with each other. Consider OVR decomposition, Equation (4), again. Since all but one class is considered to be in the negative class, the training data is likely to be imbalanced. To see why this is a problem, let us consider an extreme case where 99% of the training data is negative and only 1% of the data is positive. Then a binary classifier that always predicts the negative class would achieve 1% error. Under the framework of empirical or structural risk minimization, classifier training would tend to converge to his solution as it provides low empirical risk under 0-1 loss. Therefore a large imbalance between the size of the positive and negative sets would bias the classifier against the smaller class. So while OVR does not have any Δ entries, the average error of the binary classifiers could be high.

In the case of the AP decomposition shown in Equation (5), each individual binary classifier only has a single class serving as the positive data and another single class serving as the negative. If the overall training set was balanced between all *K *classes, then each of the binary classifiers will also be balanced and have good average error. On the other hand, the AP matrix contains many Δ entries, which is a force that increases error. As a side effect, each individual binary classifier will be faster to train compared to OVR, as the amount of data per binary classifier is reduced. This can be overshadowed by the sheer number of classifiers to train if *K *is large.

Therefore knowing which coding matrix is superior to another a priori is not possible and the choice of coding matrix **M **is application-dependent. So we must experimentally try different matrices to find which one is the best suited to the particular application.

### ncRNA dataset preparation and features

The ncRNA dataset is gathered from mirBase's collection of miRNA [19], NONCODE 3.0's collection of piRNA [20], and the remaining ncRNAs in the NONCODE 3.0 database serve as representatives of other ncRNAs. Each of these three sets is individually reduced using CD-HIT [21] to remove sequences with 80% or higher identity. This helps reduce the evolutionary correlations among the data and improves the generalization of the CRUM model that assumes an independent sample. The resulting dataset contains 9,439 miRNAs, 81,147 piRNAs, and 94,809 other ncRNAs.

In the gathered data, miRNAs are observed to be 15 ~ 33 nt long and piRNAs are observed to be 16 ~ 40 nt long. For the other ncRNAs, the training and evaluation of the McRUM does not necessarily use the entire sequence. We chose to use fragments of length 20 nt, which is in the overlapping range of the lengths between miRNAs and piRNAs, so that the fragment has the possibility of being an miRNA or piRNA had the identity of the fragment been unknown. If the other ncRNA sequence is of length longer than 20 nt, we take a random fragment of 20 nt from the sequence instead. Due to the imbalance of the dataset among the three classes, the training set is a sample of the available data. After holding out 20% of the miRNA sequences for an independent test set, we are left with 7,552 miRNAs in the training set. Therefore we sample 7,552 piRNAs and other ncRNAs each to form a balanced 1:1:1 training set. Together with the hold out of 1,887 miRNAs, the remaining 73,595 piRNAs and 87,257 other ncRNAs serve as an independent test set.

Since mature miRNAs and piRNAs lack strong secondary structures, internally the McRUM represents each ncRNA using *k*-mers, for *k *= 1, 2,..., 5. For each value of *k*, the number of occurrences of each type of *k*-mer is computed and normalized.

### Performance measures

Receiver Operating Characteristic (ROC) curve is a visualization of performance of binary classifiers at various thresholds. On the *x*-axis is the false positive rate (FPR) and on the *y*-axis is the true positive rate (TPR), which is also called sensitivity or recall. These two quantities are calculated as follows,

(15) FPR=FP/(FP+TN)

(16) TPR=TP/(TP+FN)

where *TP *is the number of true positives, *FP *is the number of false positives, *TN *is the number of true negatives, and *FN *is the number of false negatives.

**Figure 1:**
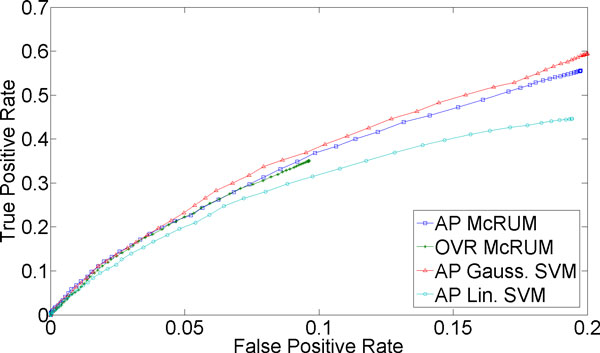
**3-fold cross-validation results for miRNA being the positive class**. The ROC curves are generated from observed FPR and TPR under varying posterior probability thresholds from 0.30 to 0.99 in increments of 0.01 for the two McRUM models (AP and OVR settings) with the Naïve decoding algorithm, and for the Gaussian and linear SVMs with all-pairs decomposition.

**Figure 2:**
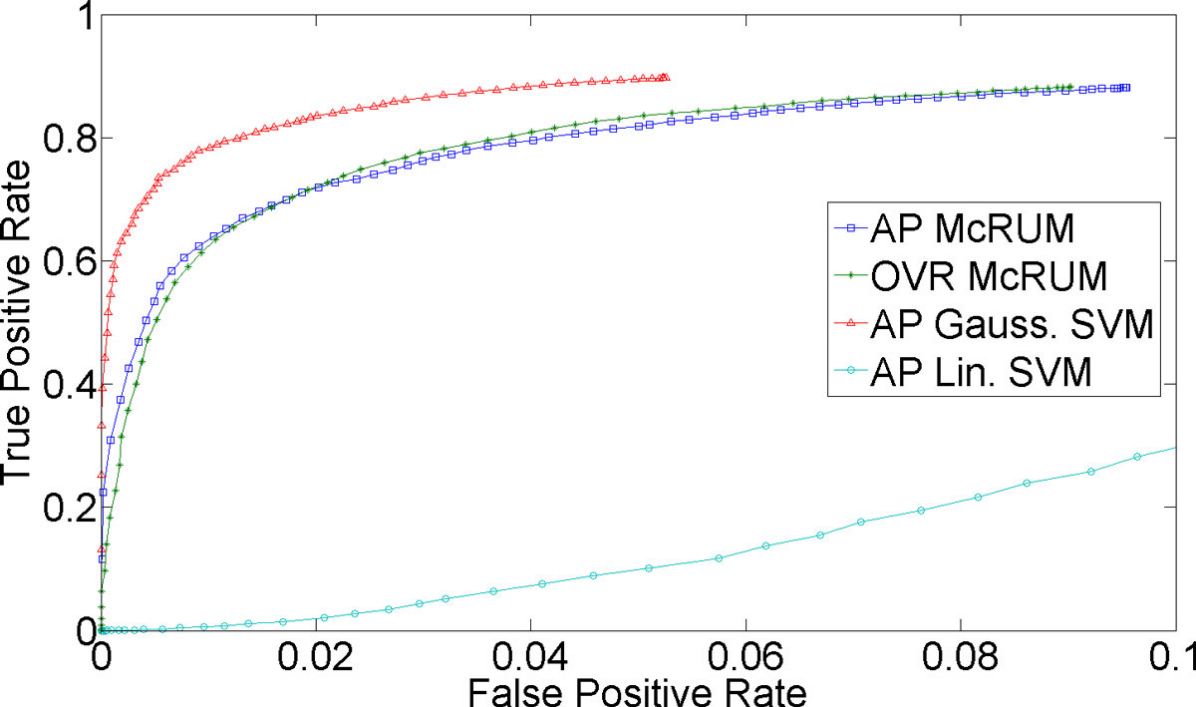
**3-fold cross-validation results for piRNA being the positive class**. The ROC curves are generated from observed FPR and TPR under varying posterior probability thresholds from 0.30 to 0.99 in increments of 0.01 for the two McRUM models (AP and OVR settings) with the Naïve decoding algorithm, and for the Gaussian and linear SVMs with all-pairs decomposition.

For classification of more than two classes, we can compute ROC curves by considering one class as the positive class and the remaining classes jointly as the negative class. For the small ncRNA experiment, we have three classes. For Figures 1(a)) and 2(a), we consider miRNA as the positive class, and piRNA and other ncRNAs jointly as the negative class. Under this setting *TP *is the count of miRNAs correctly classified, while *FP *is the number of piRNAs and other ncRNAs classified as miRNAs. *FN *is the sum of the number of miRNAs incorrectly classified and the number of miRNAs unclassified. Lastly, *TN *is the sum of the number of piRNA and other ncRNAs correctly classified, the number of piRNAs incorrectly classified as other ncRNAs, the number of other ncRNAs incorrectly classified as piRNA, and the number of piRNAs and other ncRNAs left unclassified. The piRNAs incorrectly classified as other ncRNAs, and vice versa, are counted as true negatives because they are correctly classified into the negative class and not into the positive class, miRNA. Similarly, Figures 1(b) and 2(b) are computed by considering piRNA as the positive class and miRNA and other ncRNAs jointly as the negative class, while Figures 1(c) and 2(c) are computed by considering other ncRNAs as the positive class and miRNA and piRNA jointly as the negative class.

The timing results for the Naïve and GBT decoding algorithms in the benchmark experiments were obtained using MATLAB implementations on a PC with a 2.83 GHz Intel Core 2 Quad processor and 8 GB of memory.

## Results and discussion

In this section we present two sets of experiments: benchmark experiments and small ncRNA experiments. The purpose of benchmark experiments is to assess performance of McRUM for four different decomposition settings and the two different decoding algorithms. For the experiments, we use a group of datasets from the UCI Machine Learning Repository [12], which is a collection of databases widely used for empirical analysis of algorithms in the machine learning community. The performance of McRUM with different settings is presented in terms of prediction accuracy. The small ncRNA experiments are conducted to validate the use of McRUM on a larger scale problem. Through the benchmark experiments, Naïve decoding algorithm is proven to be much more computationally efficient than the GBT algorithm with its AP and OVR performances being close to the GBT results. Therefore, in the ncRNA experiments, only the Naïve McRUMs with AP and OVR settings are tested and the ROC analysis is used to illustrate how the performance of selected McRUM models changes at various decision thresholds.

For both sets of experiments, we also run the multiclass SVM implemented in LIBSVM[22] to illustrate McRUM's performance relative to SVM-based approaches. The LIBSVM implementation uses the all-pairs decomposition, Platt's method to generate probabilities from the individual binary SVMs, and their own algorithm for aggregating the all-pairs binary results to multiclass posterior probabilities. The multiclass SVM implementation is freely available at [23].

### Benchmark experiments

For the experiments, we try the McRUM on five small datasets from the UCI Machine Learning Repository website [12] using the binary CRUM classifiers with the Gaussian kernel. The kernel width γ and model complexity *M *is chosen via K-means clustering of the unlabeled training dataset [9]. For a range of values of *M*, K-means clustering is applied to group the unlabeled training dataset into *M *clusters and the Akaike Information Criterion (AIC) is computed by giving the clustering results a probabilistic interpretation. The number of clusters with the best AIC value is selected for *M*. Furthermore, we set γ = (2*d*^2^)^-1^, where *d *is the maximum distance between cluster centers obtained by the K-means clustering with K = *M*. Using K-means clustering to set parameters as described above has clear computational advantages, which can be critical when training is performed on large datasets. The same γ and *M *are used for all the individual binary classifiers per dataset.

Throughout the benchmark experiments, we consider the following decompositions: (i) all-pairs (AP), (ii) one-versus-rest (OVR), (iii) random dense, and (iv) random sparse. Random coding matrices **M **are generated with and without using Δ symbols for the random sparse and random dense cases, respectively. For each random type, 100 random **M **are generated and the **M **with the smallest minimum distance among its rows is chosen. Controlling the number of columns in the random sparse matrix, we can aim to create a decomposition that is a compromise between AP and OVR. This is useful should the number of classes *K *be large and AP impractical, while still retaining some of the reduced training set benefits per binary classifier. The details of the different decompositions are given in the Methods section.

The class label is assigned based on which class has the largest posterior probability, as determined by the Naïve and generalized Bradley-Terry (GBT) decoding algorithms [11]. Since the GBT algorithm is not guaranteed to converge, a maximum of 1000 iterations of the algorithm is imposed. We first examine results using cross-validation.

#### Wine dataset

The three-class wine dataset contains 178 instances [12,24]. The objective is to determine the origin of the wines based on 13 physicochemical measurements. The number of binary classifiers for AP and OVR are both three and so we set the dense and sparse decompositions to also use three classifiers. The mean accuracy and its standard deviation results in Table 1 are computed via 10-fold cross-validation. The AP and OVR decompositions give nearly the same results regardless of using the Naïve or GBT algorithms. There is a significant reduction in accuracy for the Naïve algorithm for the random dense and sparse cases, but the GBT results remain close to the Naïve algorithm's AP and OVR performance.

**Table 1:** McRUM results on wine dataset using 10-fold cross-validation

	Naïve		GBT	
	
	Train Acc	Test Acc	Train Acc	Test Acc
AP	99.44 (0.20)	97.78 (2.87)	99.44 (0.20)	97.78 (2.87)

OVR	99.44 (0.20)	97.75 (3.92)	99.44 (0.20)	97.75 (3.92)

Dense	84.40 (1.09)	83.63 (6.99)	99.44 (0.20)	98.30 (2.74)

Sparse	91.01 (1.76)	90.00 (6.31)	99.50 (0.26)	97.22 (4.72)

Train/Test Acc is the mean accuracy on the training/test dataset. The standard deviation is shown in the parenthesis. (AP: all-pairs, OVR: one-versus-rest, Dense: random dense, Sparse: random sparse)

The prediction running-times are also measured for the AP and OVR decompositions where the Naïve and GBT algorithms show comparable predictive performance. As shown in Table 2, Naïve algorithm achieves a three orders of magnitude speed-up compared to GBT algorithm for both AP and OVR decomposition.

**Table 2:** Prediction time of McRUM on benchmark datasets (in seconds)

	Naïve		GBT	
	
	AP	OVR	AP	OVR
wine	0.001868	0.001662	7.373405	1.073139

	(0.000206)	(0.000164)	(1.182947)	(0.197278)

iris	0.001409	0.001376	6.625228	2.908828

	(0.000139)	(0.000141)	(0.678363)	(0.834858)

yeast	0.040737	0.0492920	2722.317544	2795.766035

	(0.000534)	(0.001219)	(117.545388)	(139.314294)

thyroid	0.131689	0.123968	939.692526	179.426632

satellite	0.239550	0.139304	10612.598301	2816.632703

We provide the prediction time only for AP and OVR cases since their predictive performances are competitive to each other for all benchmark datasets while the performances for random decompositions are much lower than the AP and OVR cases with Naïve algorithm. The prediction time is averaged over 10-fold cross-validation for the first three datasets while it is estimated once for the last two datasets as their explicit partitioning of training and test sets were given. Included in the parenthesis is the standard deviation of the prediction time. (AP: all-pairs, OVR: one-versus-rest)

We observed the mean accuracies of 99.69% (std = 0.33) and 98.89% (std = 2.34) from Gaussian SVM for the training and test set, respectively, which is comparable to the AP and OVR McRUM results of 99.44% (std = 0.20) and 97.78% (std = 2.87). In addition, the best mean accuracy reported for a 10-fold cross-validation using a multiclass RVM for this wine dataset is 96.24% [25], which, considering the standard deviation, is also comparable to the best achieved here using the McRUM.

#### Iris dataset

Table 3 gives the 10-fold cross-validation results on the three-class iris dataset, a classic machine learning and statistics benchmark. The problem is to classify flowers of three species of the genus *Iris *based on four physical measurements from a sample of 150 flowers [12,26]. We see similar results as earlier, where the Naïve and GBT accuracies are nearly the same and the performance of Naïve algorithm drops severely under the random decompositions. Three binary classifiers are used in all cases. As for prediction running-times, as shown in Table 2, there is a three order of magnitude speed-up with the Naïve compared to the GBT algorithm with minimal impact to predictive performance, as also seen with the wine dataset results previously.

**Table 3:** McRUM results on iris dataset using 10-fold cross-validation

	Naïve		GBT	
	
	Train Acc	Test Acc	Train Acc	Test Acc
AP	97.56 (0.70)	96.00 (5.62)	97.56 (0.70)	96.00 (5.62)

OVR	97.85 (0.82)	96.67 (5.67)	97.85 (0.82)	96.67 (5.67)

Dense	67.56 (2.88)	68.00 (16.27)	97.70 (0.74)	96.00 (6.44)

Sparse	66.67 (1.16)	66.67 (10.42)	97.78 (0.92)	95.33 (5.49)

Train/Test Acc is the mean accuracy on the training/test dataset. The standard deviation is shown in the parenthesis. (AP: all-pairs, OVR: one-versus-rest, Dense: random dense, Sparse: random sparse)

The mean accuracies on the training and test set we observed from Gaussian SVM are 97.85% (std = 0.65) and 96.67% (std = 3.51). The best mean accuracy reported for a 10-fold cross-validation using a multiclass RVM for this iris dataset is 93.87% [25]. Again, both multiclass SVM and RVM results are comparable to the best achieved here with McRUM (97.85% (std = 0.82) and 96.67% (std = 5.67)).

#### Yeast dataset

Table 4 gives the 10-fold cross-validation for the 10-class yeast dataset that contains 1,484 proteins. The goal is to predict the cellular localization site of a protein based on eight sequence-related measures [2,12]. 18 binary classifiers are used for the random sparse decomposition to achieve a compromise between AP and OVR. For the random dense decomposition, we used 34 classifiers as a sample of the columns of the "complete dense" decomposition that contains all possible partitions of the data into positive and negative classes. Only the mean accuracy on the test partitions are given, as the dataset is large enough that computing the accuracy of the training partitions is prohibited by the complexity of the GBT algorithm. The extra effort of the GBT algorithm is required in the random decompositions cases where the Naïve results are dismal. The results for AP and OVR, however, are comparable between Naïve and GBT.

**Table 4:** McRUM results on yeast dataset using 10-fold cross-validation

	Naïve Test Acc	GBT Test Acc
	
AP	59.43 (6.27)	59.10 (6.21)
OVR	58.90 (5.29)	59.51 (4.02)

Dense	3.43 (1.49)	57.75 (3.11)

Sparse	2.02 (1.62)	59.23 (2.79)

Test Acc is the mean accuracy on the test dataset. The standard deviation is shown in the parenthesis. Note that, due to the large dataset size and the high computational complexity of the GBT algorithm, the mean accuracy on the training partitions cannot be provided. (AP: all-pairs, OVR: one-versus-rest, Dense: random dense, Sparse:random sparse)

For this dataset, Gaussian SVM shows 60.85% accuracy with standard deviation of 4.08. Best results from AP McRUM (59.43% (std = 6.27)) and OVR McRUM (59.51% (std = 4.02) are again comparable to the SVM results considering the standard deviation. The results from both McRUM and SVM are an improvement over the 56.5% achieved in the dataset's original analysis using PSORT [2] and are comparable to the 59.51% (standard deviation of 5.49) obtained by k-NN with PSORT II on this yeast dataset [3].

The prediction running-times in Table 2 show five orders of magnitude speed-up for the AP and OVR McRUMs using the Naïve algorithm over the GBT algorithm. This very significant speed-up is achieved with minimal impact to the predictive performance, as discussed above.

#### Thyroid disease dataset

The results in Table 5 are from the three-class thyroid dataset that has 3,772 and 3,428 training and testing instances, respectively. The problem is to determine if a patient has hypothyroid based on 21 attributes [12,27,28]. The number of binary classifiers for AP and OVR are both three and so we set the dense and sparse decompositions to also use three classifiers. Regardless of the posterior decoding algorithm, the AP decomposition performs with the best accuracy. The Naïve and GBT algorithms perform similarly on all but the sparse decomposition. It appears the independent binary classifiers assumption fails in the sparse case and the Naïve algorithm does not approximate the posteriors for class 2 and 3 as well as the GBT algorithm does. The SVM results we obtained for this dataset are 96.29% and 94.37% for training and test accuracy, respectively. The AP results from the McRUM (98.44% and 97.22%) are slightly better.

**Table 5:** McRUM results on thyroid training and test datasets

	Naïve		GBT	
	
	Train Acc	Test Acc	Train Acc	Test Acc
AP	98.44	97.29	97.93	97.11

OVR	95.47	95.22	95.52	95.04

Dense	92.47	92.71	94.38	93.90

Sparse	24.68	25.50	97.11	96.18

Train/Test Acc is the accuracy on the training/test dataset. Note that an explicit partitioning of the data into training and test sets were provided. Therefore, cross-validation experiment was not performed and, as a result, no information on standard deviation is available. (AP: all-pairs, OVR: one-versus-rest, Dense: random dense, Sparse: random sparse)

As shown in Table 2, under the AP decomposition, it takes about 0.132 seconds to compute the predictions for the entire test set using the Naïve algorithm. On the other hand, with a maximum of 1,000 iterations per datapoint, the GBT algorithm takes about 934.693 seconds, almost four orders of magnitude longer than the Naïve algorithm for nearly the same predictive accuracy. In the case of OVR, the predictions times for the test set are 0.124 and 179.427 seconds for Naïve and GBT algorithms, respectively. The OVR decomposition appears to be an easier problem for the GBT algorithm to solve than the AP decomposition despite using the same number of binary classifiers.

#### Landsat satellite (statlog) dataset

Table 6 gives the results on a satellite image (statlog) dataset that has six classes with 4,435 and 2,000 training and testing instances, respectively. The goal is to interpret a scene based on 36 multi-spectral values [12,27]. Here for both training and test accuracy, both the AP and OVR under Naïve and GBT decoding perform at about the same level. OVR uses 6 binary classifiers, while AP uses 15. To strike a halfway point between the two, the random decompositions use 10 binary classifiers. However, for these random decompositions, the Naïve algorithm is outperformed by GBT. The SVM results we obtained for this dataset are 98.35% and 91.50% for training and test accuracy, respectively. For training set, SVM is superior to AP and OVR McRUMs (89.85% and 89.76%) and both McRUMs' performance on test set (88.05% and 87.70%) is also slightly worse than the SVM.

**Table 6:** McRUM results on satellite image training and test datasets

	Naïve		GBT	
	
	Train Acc	Test Acc	Train Acc	Test Acc
AP	89.85	88.05	89.76	87.70

OVR	89.56	87.65	89.58	87.50

Dense	10.60	11.85	80.56	77.95

Sparse	23.40	23.50	85.73	84.55

Train/Test Acc is the accuracy on the training/test dataset. Note that an explicit partitioning of the data into training and test sets was provided. Therefore, cross-validation experiment was not performed and, as a result, no information on standard deviation is available. (AP: all-pairs, OVR: one-versus-rest, Dense: random dense, Sparse: random sparse)

Table 2 shows that, for the test set, it takes about 0.240 and 10,612.598 seconds for the Naïve and GBT algorithms to compute the predictions under the AP decomposition, respectively. For the OVR decomposition, the prediction times are 0.139 and 2,816.633 seconds for Naïve and GBT algorithms, respectively. Again the OVR is the faster of the two decompositions, partly because of the reduced number of binary classifiers.

### Small ncRNA experiments

To validate the McRUM on a larger scale problem and to explore its use for the task of NGS data analysis, we investigated the classification of mature miRNAs and piRNAs from other ncRNAs. This is a problem of interest in the analysis of small RNA sequencing (RNA-seq) data. Further details of the dataset and sequence features used by the McRUM are given in the Methods section. For this experiment, two McRUM models are used for the AP and OVR settings using the Naïve decoding algorithm, and their performance is illustrated relative to the Gaussian and linear multiclass SVMs.

#### Cross-validation experiments

Figures 1 through 3 show ROC curves under 3-fold cross-validation experiments. Note that, because ROC curves are defined for binary classification, each ROC curve considers one of the three classes as the positive class, while considering the remaining two classes jointly as the negative class. For example, Figure 1 considers miRNA as the positive class and piRNA and other ncRNA as the negative class. The details of the computation of the ROC curves are given in the Methods section. The following results are obtained using the best observed model complexity, *M *= 600, for the underlying binary CRUM models using the Gaussian kernel. The kernel width γ is chosen using K-means clustering of the unlabeled training dataset with the selected *M*. For multiclass SVM, we trained Gaussian SVMs and linear SVMs where, on average, about 3,725 support vectors were used in each binary Gaussian SVM and about 4,048 for each binary linear SVM.

Figure 1 suggests that classifying mature miRNA is a very difficult problem, as even at the highest observed FPR (about 0.2), the TPR only approaches 0.6 for both McRUM and SVM. There is no clear superior decomposition for McRUM. However, the OVR McRUM occupies a narrower range of FPRs and cannot achieve as high a TPR as the AP case. Although both versions of McRUM perform slightly better than linear SVM and slightly worse than Gaussian SVM, the performances are all very similar. Based on these results and those given below, it is seen that mature miRNAs often get confused with ncRNAs other than piRNA.

In contrast to miRNA, Figure 2 suggests that the classification of piRNA is a much easier problem. The TPR approaches 0.9 even at a low FPR of 0.09 for McRUM and 0.5 for Gaussian SVM. The ROC curves for AP and OVR McRUMs are comparable while Gaussian SVM shows higher performance and linear SVM works very poorly. The poor performance of linear SVM may be due to the non-linear nature of the problem. Although the Gaussian SVM shows superior performance, both AP and OVR McRUMs also achieve similar level of TPR by sacrificing FPR slightly. Furthermore, given more compact models and better computational efficiency of binary CRUM over SVM [6], McRUM can still be a favorable choice for large-scale prediction problems.

**Figure 3:**
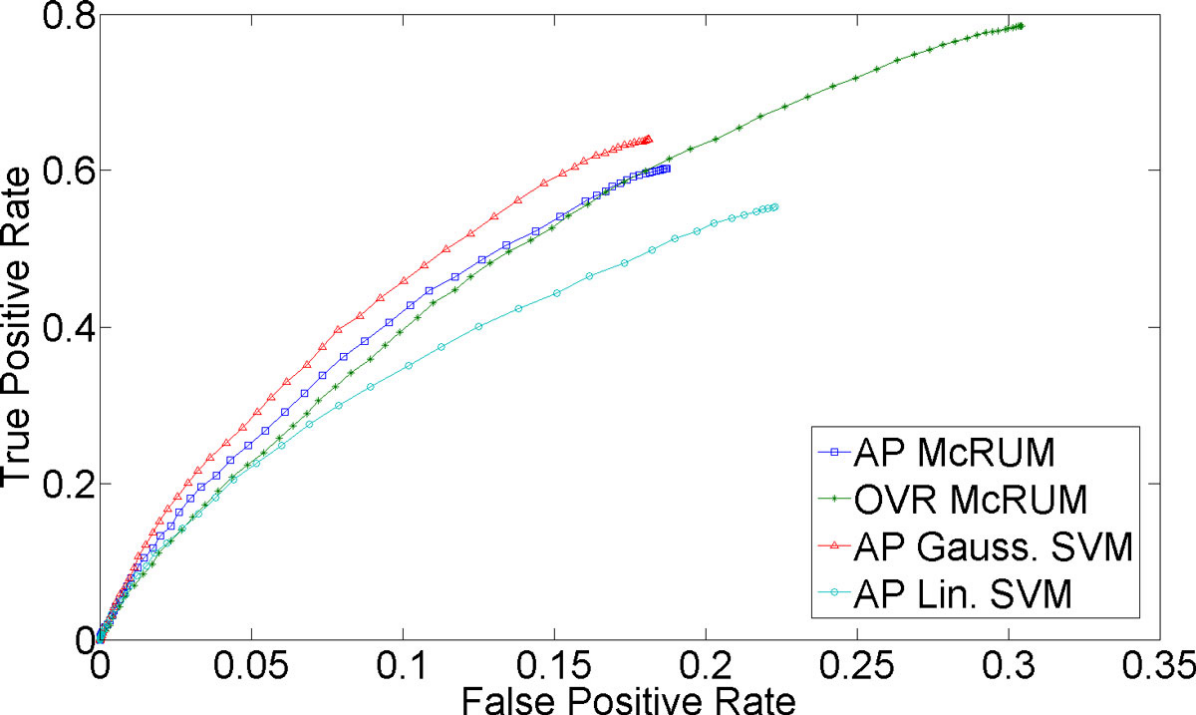
**3-fold cross-validation results for non-miRNA and non-piRNA being the positive class**. The ROC curves are generated from observed FPR and TPR under varying posterior probability thresholds from 0.30 to 0.99 in increments of 0.01 for the two McRUM models (AP and OVR settings) with the Naïve decoding algorithm, and for the Gaussian and linear SVMs with all-pairs decomposition.

Finally, Figure 3 shows that discriminating the class consisting of other ncRNAs is also difficult, but not as difficult as the miRNA case. Again, the performance suffers due to the difficulty of discriminating miRNA from other ncRNAs. While linear SVM shows poor performance, in the region of overlapping FPRs, the ROC curves for Gaussian SVM and both OVR and AP McRUMs are comparable. However, the OVR McRUM has a wider FPR range, allowing it to achieve a high TPR of about 0.8 at an FPR of about 0.3, that the AP McRUM and Gaussian SVM cannot obtain.

In Additional file 1, we provide a plot showing the fraction of the validation set left unclassified for the AP and OVR McRUMs and the Gaussian and linear SVMs at different posterior probability threshold values. In summary, linear SVM is uniformly worse than the others, AP McRUM and Gaussian SVM have almost the same fraction of unclassified sequences, and OVR McRUM shows the smallest number of unclassified sequences across a wide range of threshold values.

#### Independent test experiments

**Figure 4:**
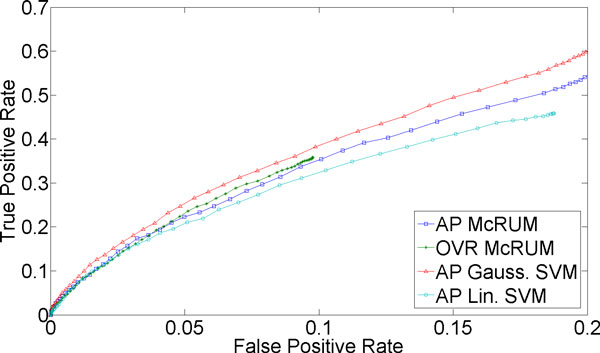
**Evaluation results on independent test set for miRNA being the positive class**. The ROC curves are generated from observed FPR and TPR under varying posterior probability thresholds from 0.30 to 0.99 in increments of 0.01 for the two McRUM models (AP and OVR settings) with the Naïve decoding algorithm, and for the Gaussian and linear SVMs with all-pairs decomposition.

**Figure 5:**
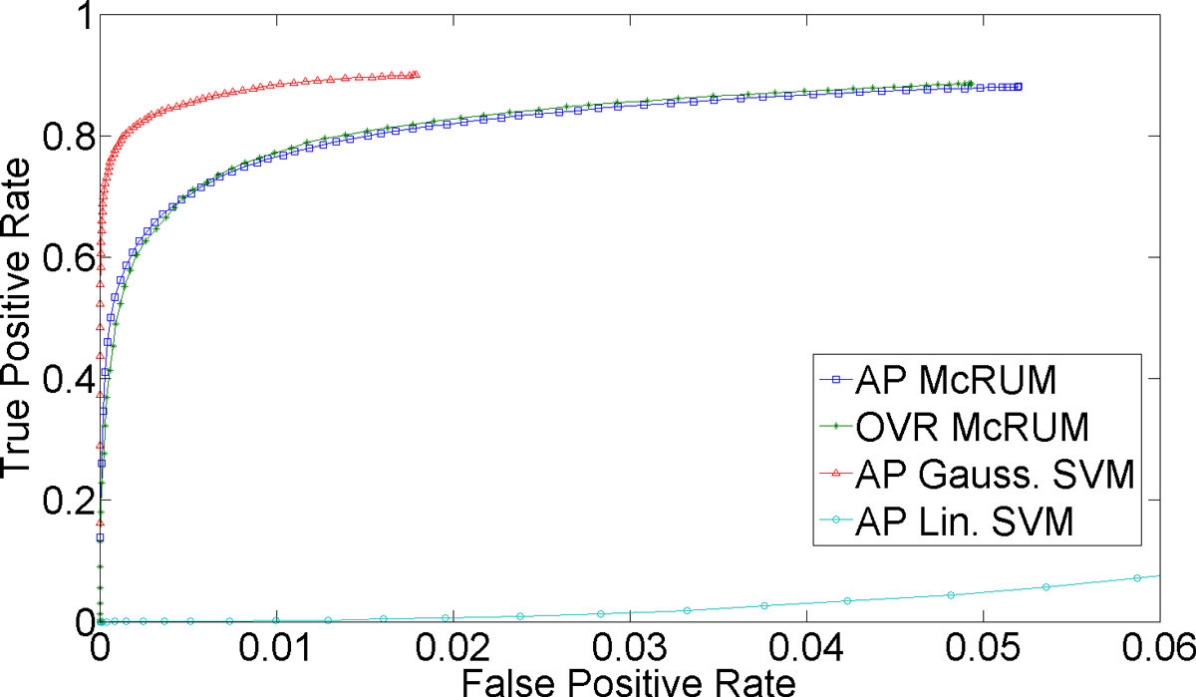
**Evaluation results on independent test set for piRNA being the positive class**. The ROC curves are generated from observed FPR and TPR under varying posterior probability thresholds from 0.30 to 0.99 in increments of 0.01 for the two McRUM models (AP and OVR settings) with the Naïve decoding algorithm, and for the Gaussian and linear SVMs with all-pairs decomposition.

**Figure 6:**
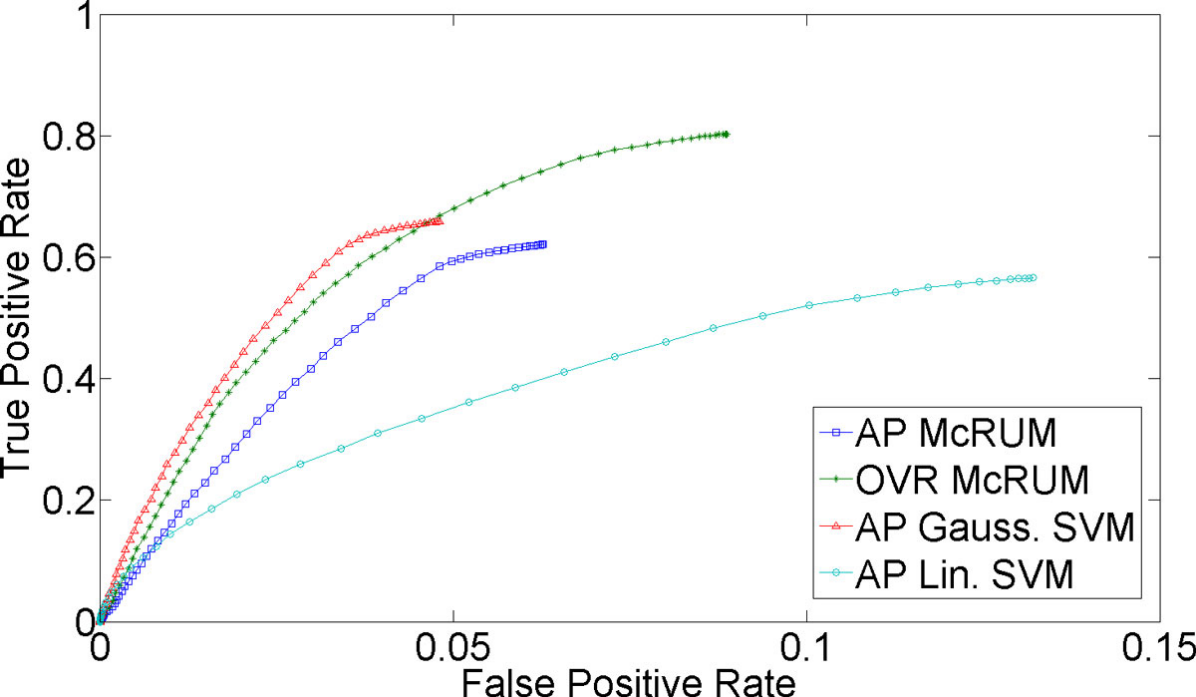
**Evaluation results on independent test set for non-miRNA and non-piRNA being the positive class**. The ROC curves are generated from observed FPR and TPR under varying posterior probability thresholds from 0.30 to 0.99 in increments of 0.01 for the two McRUM models (AP and OVR settings) with the Naïve decoding algorithm, and for the Gaussian and linear SVMs with all-pairs decomposition.

Further evaluation of the McRUM and the multiclass SVMs on a larger, independent dataset was also conducted and the ROC curves are given in Figures 4 through 6. McRUMs are trained with *M *= 600 and SVMs are trained with about 5,410 support vectors on average for each binary Gaussian SVM and about 6,034 for each binary linear SVM using the entire training dataset used in the cross-validation experiment. Figure 4 reiterates that the miRNA case is very difficult, showing results similar to the cross-validation experiment. For the piRNA case shown in Figure 5, we again see very good performance from Gaussian SVM and both the AP and OVR McRUMs, and extremely poor performance from linear SVM. Note that the scale of the FPR axis is very small. As described above, AP and OVR McRUMs can achieve the similar level of TPR as Gaussian SVM by sacrificing FPR slightly, about 0.03. In addition, the McRUMs are computationally more efficient than the multiclass SVMs since the binary CRUM is less expensive computationally than the binary SVM [6]. To compute the class posterior probabilities of the entire test dataset, the SVMs take about 2.5 to 3 times longer than the McRUMs even though the SVM implementation is in faster compiled C++, whereas McRUM is implemented in MATLAB, a slower interpreted language. (OVR McRUM takes 3,203.35 sec, AP McRUM 3,429.91 sec, Gaussian SVM 11,015.29 sec, and linear SVM 8,444.61 sec.) Lastly in Figure 6, we see the remaining ncRNAs showing good results with Gaussian SVM and the OVR McRUM that are slightly better than the AP McRUM.

In Additional file 2, we provide a plot showing the fraction of the test set left unclassified for the AP and OVR McRUMs and the Gaussian and linear SVMs at different posterior probability threshold values. The results are very similar to those obtained for the cross-validation experiments shown in Additional file 1.

Recently, a Fisher Linear Discriminant (FLD) based classifier called piRNApredictior has been proposed for binary piRNA classification by Zhang et al. [29]. We have downloaded the script from their website [30] and trained it with the training dataset used in [29] (FLD_1) and also with our own training dataset (FLD_2). Then the resulting classifiers were evaluated on our test dataset. For the prediction step, both FLD-based classifiers constrain the input sequence being at least 25 nt. We removed this constraint in the script as our test dataset contains many ncRNAs (both piRNAs and non-piRNAs) shorter than 25 nt.

**Figure 7:**
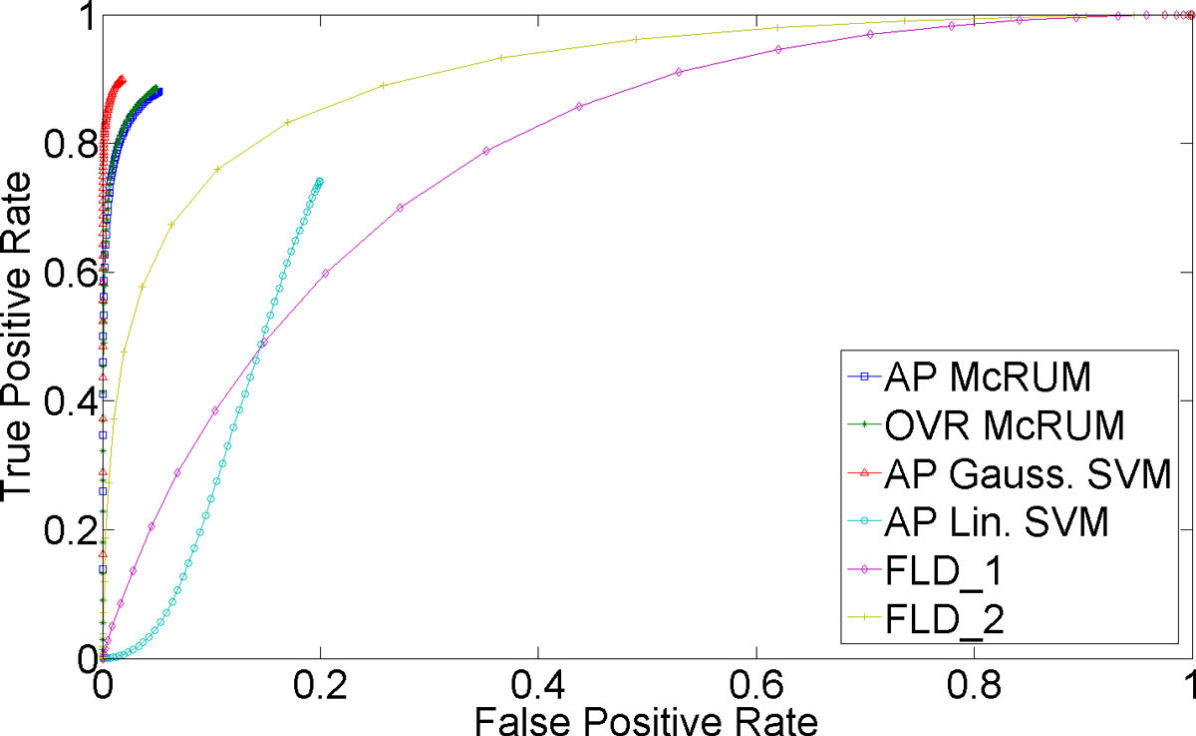
**piRNA prediction results**. The ROC curves for FLD-based classifiers are generated from observed prediction results on our test dataset with the threshold varying from -0.2 to 0.46 in increments of 0.001. (-0.2 and 0.46 are the lower and upper bounds of the observed predicted values of FLD-based classifiers.) FLD_1 classifier was trained with the dataset used in [29] while FLD_2 used our training dataset. McRUM and SVM results are the same as shown in Figure 5.)

**Figure 8:**
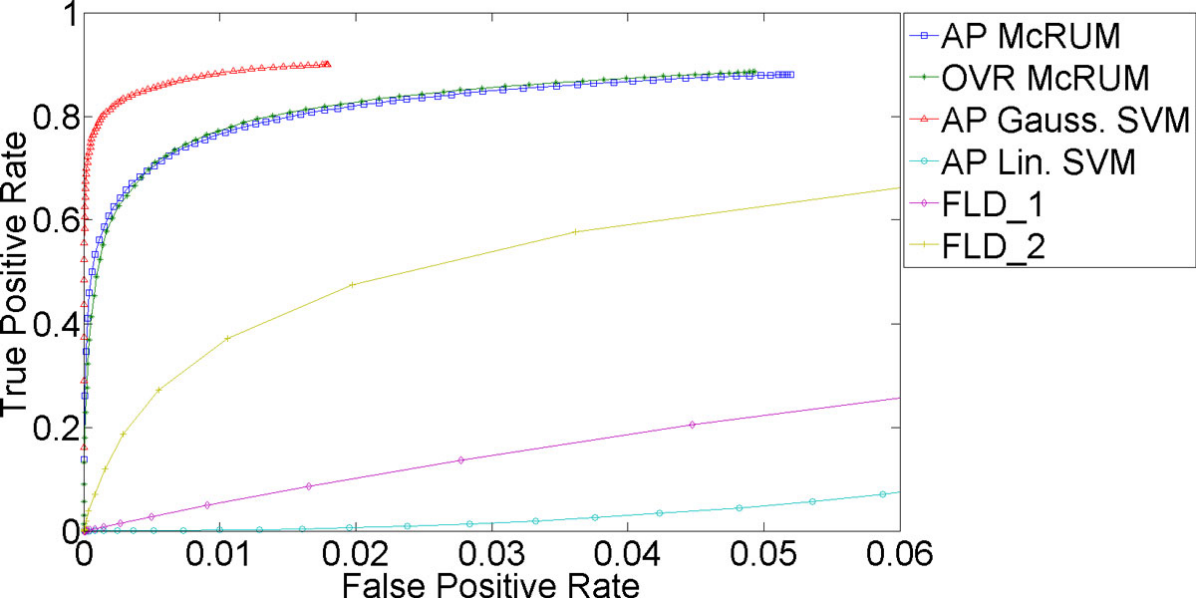
**piRNA prediction results**. The ROC curves in Figure 7 are zoomed in to match the FPR range shown in Figure 5. The ROC curves for FLD-based classifiers are generated from observed prediction results on our test dataset with the threshold varying from -0.2 to 0.46 in increments of 0.001. (-0.2 and 0.46 are the lower and upper bounds of the observed predicted values of FLD-based classifiers.) .) FLD_1 classifier was trained with the dataset used in [29] while FLD_2 used our training dataset. McRUM and SVM results are the same as shown in Figure 5.)

The ROC curves generated from observed results of the FLD-based classifiers are presented in Figures 7 and 8 along with the ROC curves for McRUM and SVM results. Both AP and OVR McRUMs and Gaussian SVM clearly show superior predictive performance over the FLD-based classifiers. One reason for the lower performance of FLD-based classifiers can be the possible nonlinear boundary between the classes of ncRNAs under the features considered. The nonlinearity of the problem is also evident from the extremely poor performance of linear SVM.

Note that about 99% of the sequences in the positive training dataset for FLD_1 are from NONCODE 2.0's collection of piRNA. Our positive test dataset is gathered from a later version of NONCODE database and, as a result, 98.57% of the sequences in our positive test dataset are already included in the positive training dataset used for FLD_1. Therefore the prediction results may be biased in favor of FLD_1. Then, FLD_2 showing better performance than FLD_1 may seem contradictory when the training set used for FLD_2 is independent of the test set. It can be because FLD_1 is not specifically trained on the ncRNAs shorter than 25 nt. The training dataset for FLD_1 contains about 4.67% ncRNAs shorter than 25 nt while our training dataset used for FLD_2 contains 66.41% sequences shorter than 25 nt. In the test dataset, 55.93% of the sequences in the test dataset are shorter than 25 nt, for which correct prediction can be hard for FLD_1.

## Conclusions

In this study, the binary CRUM model is generalized to the multiclass setting via ECOC framework. The probabilistic nature of the binary CRUM is preserved using either the GBT or the proposed linear-time decoding algorithms. The proposed linear-time algorithm allows for efficient application to large-scale prediction settings, where the GBT algorithm's complexity is prohibitive, while still maintaining comparable predictive performance under certain decompositions of the given multiclass problems, as evidenced by the benchmark experiments. The applicability of the McRUM to larger scale problems is demonstrated by an analysis of small ncRNA sequences. The results demonstrate that McRUM can be an advantageous solution to resolve multiclass problems especially when applied to large datasets.

The preliminary results on small ncRNA classification presented in this paper demonstrate that the McRUM has potential in addressing the problem of classifying small ncRNAs. In this study, we restricted the length of the other ncRNA fragments to be maximum of 20 nt, but we plan to conduct further experiments with various lengths of fragments. We also plan to include short byproducts of small RNA biogenesis, such as miRNA*, in the class of other ncRNAs. In the future, we will also extend the current study by including other classes of small ncRNAs and optimizing the use of the McRUM for large-scale datasets such as those generated by NGS sequencing projects. Features other than the simple k-mers will be considered to improve the predictive performance, especially for classifying the mature miRNAs. Finally, the interesting preliminary results obtained by the multiclass Gaussian SVM on the problem of small ncRNA classification show that it could be an advantageous alternative to McRUM on smaller datasets and thus we intend to develop in tandem both classifiers for further experiments. The resulting small ncRNA classifiers will be integrated into a combined prediction tool that will offer both the multiclass SVM and McRUM options providing more alternative choices to users.

## Competing interests

The authors declare that they have no competing interests.

## Authors' contributions

MM implemented the McRUM and performed the experiments. All authors participated in the design of the study, development of CRUM and the data analyses. KB and GP supervised and coordinated the whole research work. All authors have read, revised and approved the final manuscript.

## Acknowledgements

This work is supported in part by NIH Grants from the National Institute of General Medical Sciences (P20GM103516 and P20GM103466). The paper's contents are solely the responsibility of the authors and do not necessarily represent the official views of the NIH.

## Supplementary Material

Additional file 1 **The fraction of unclassified sequences for cross-validation experiment**. It is a figure in tif format named 'MenorBaekPoisson-Figure S1.tif' showing the fraction of the validation set left unclassified for the AP and OVR McRUMs and the Gaussian and linear SVMs at different posterior probability threshold values. Click here for file

Additional file 2 **The fraction of unclassified sequences for independent test experiment**. It is a figure in tif format named 'MenorBaekPoisson-Figure S2.tif' showing the fraction of the test set left unclassified for the AP and OVR McRUMs and the Gaussian and linear SVMs at different posterior probability threshold values. Click here for file

## Declarations

The publication costs for this article were funded by grant number P20GM103516 from the National Institute of General Medical Sciences, of the National Institutes of Health.

This article has been published as part of *BMC Genomics *Volume 14 Supplement 2, 2013: Selected articles from ISCB-Asia 2012. The full contents of the supplement are available online at http://www.biomedcentral.com/bmcgenomics/supplements/14/S2.
