# Supplementary figures and images for: Multiclass relevance units machine: benchmark evaluation and application to small ncRNA discovery
Source: BMC Genomics. 2013 Feb 15;14(Suppl 2):S6. doi: 10.1186/1471-2164-14-S2-S6 (PMC3582431; doi:10.1186/1471-2164-14-S2-S6)

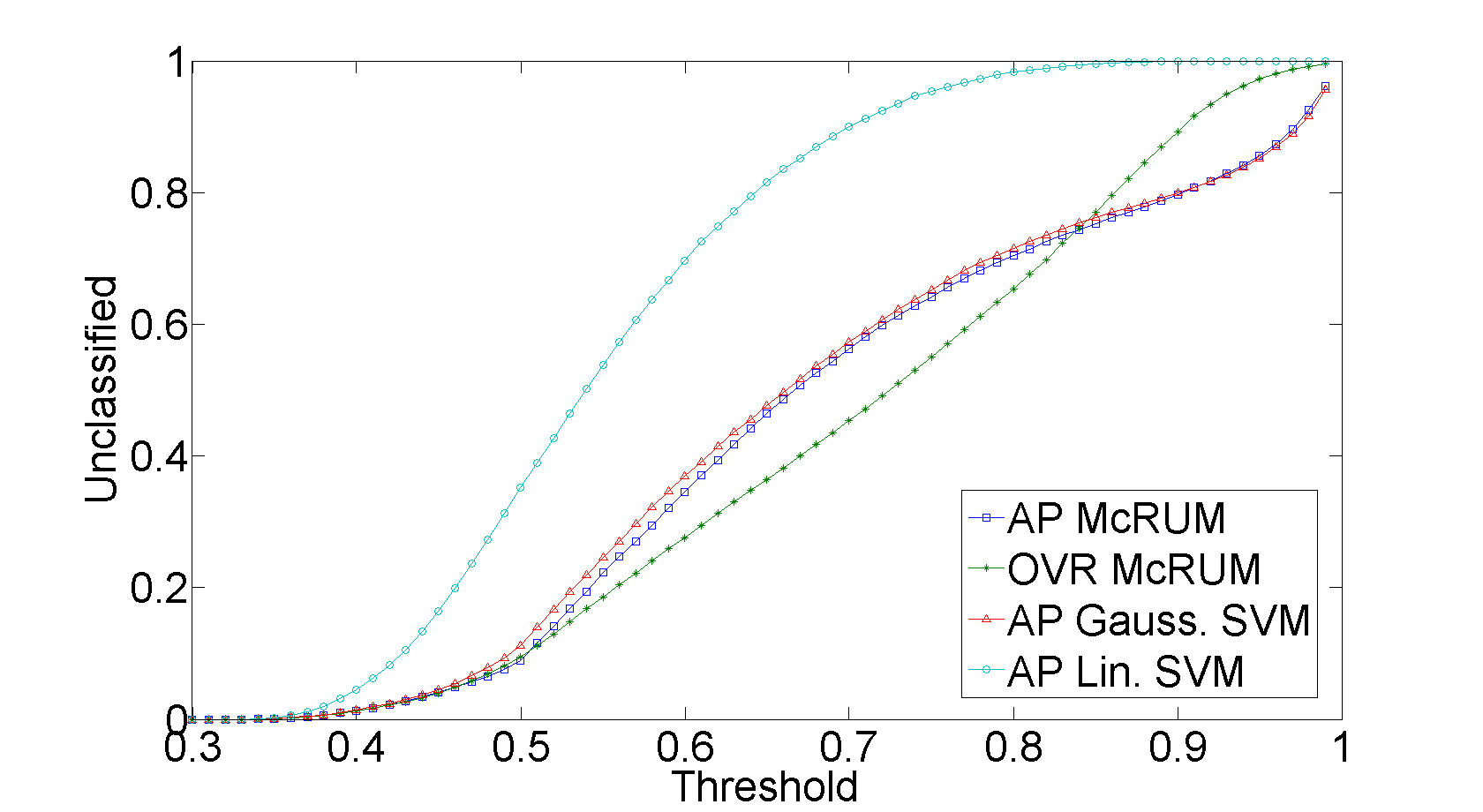

Supplement: Additional file 1 — The fraction of unclassified sequences for cross-validation experiment. It is a figure in tif format named 'MenorBaekPoisson-Figure S1.tif' showing the fraction of the validation set left unclassified for the AP and OVR McRUMs and the Gaussian and linear SVMs at different posterior probability threshold values. [file 1471-2164-14-S2-S6-S1.TIF]

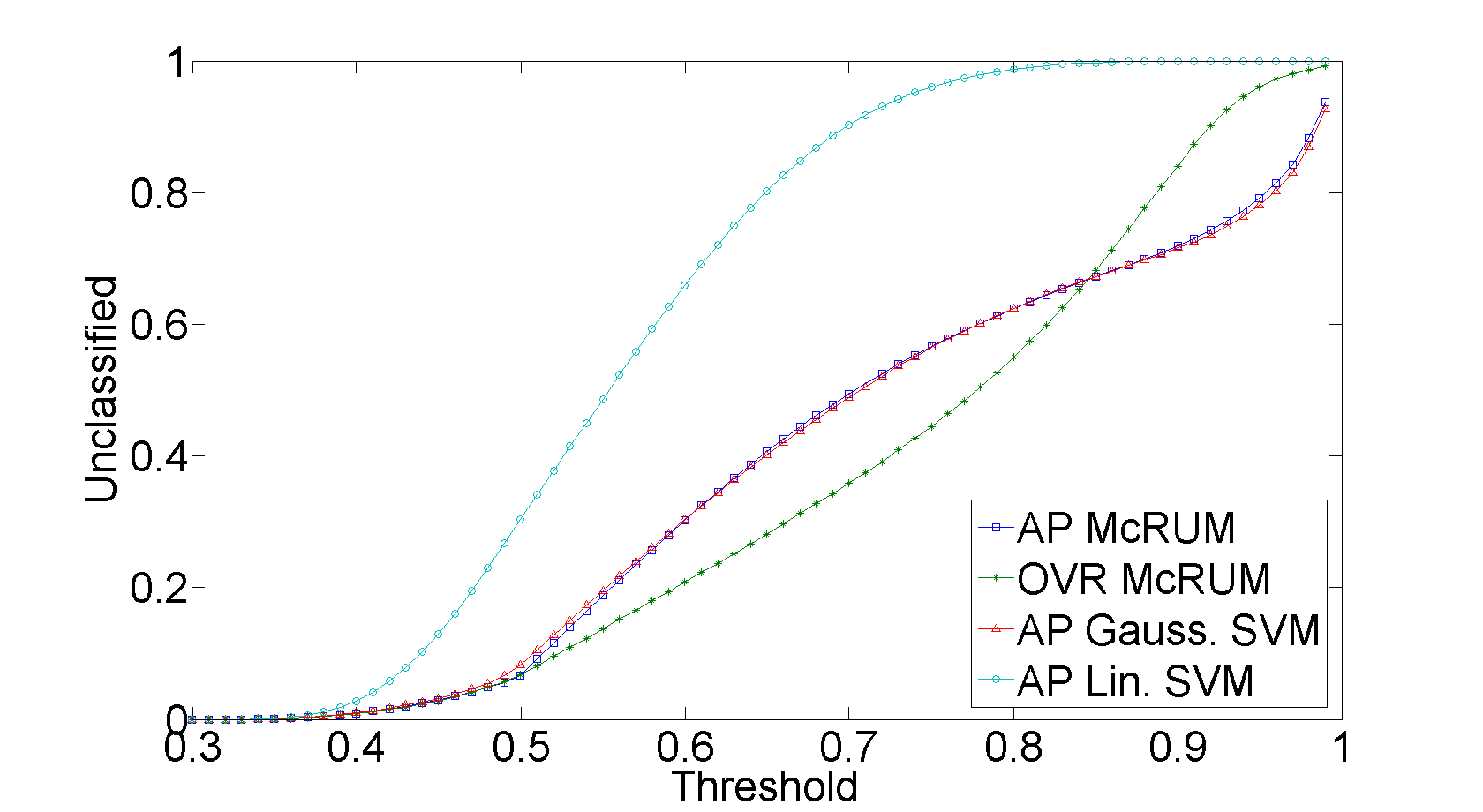

Supplement: Additional file 2 — The fraction of unclassified sequences for independent test experiment. It is a figure in tif format named 'MenorBaekPoisson-Figure S2.tif' showing the fraction of the test set left unclassified for the AP and OVR McRUMs and the Gaussian and linear SVMs at different posterior probability threshold values. [file 1471-2164-14-S2-S6-S2.TIF]
